# Copy number variants selected during pig domestication inferred from whole genome resequencing

**DOI:** 10.3389/fvets.2024.1364267

**Published:** 2024-03-05

**Authors:** Wei Zhang, Chengliang Xu, Mei Zhou, Linqing Liu, Zelan Ni, Shiguang Su, Chonglong Wang

**Affiliations:** ^1^Institute of Animal Husbandry and Veterinary Medicine, Anhui Academy of Agricultural Sciences, Anhui Provincial Breeding Pig Genetic Evaluation Center, Key Laboratory of Pig Molecular Quantitative Genetics of Anhui Academy of Agricultural Sciences, Anhui Provincial Key Laboratory of Livestock and Poultry Product Safety Engineering, Hefei, China; ^2^Anhui Provincial Livestock and Poultry Genetic Resources Conservation Center, Hefei, China

**Keywords:** Wanbei pig, Asian wild boar, copy number variation (CNV), selection signature, whole genome resequencing

## Abstract

Over extended periods of natural and artificial selection, China has developed numerous exceptional pig breeds. Deciphering the germplasm characteristics of these breeds is crucial for their preservation and utilization. While many studies have employed single nucleotide polymorphism (SNP) analysis to investigate the local pig germplasm characteristics, copy number variation (CNV), another significant type of genetic variation, has been less explored in understanding pig resources. In this study, we examined the CNVs of 18 Wanbei pigs (WBP) using whole genome resequencing data with an average depth of 12.61. We identified a total of 8,783 CNVs (~30.07 Mb, 1.20% of the pig genome) in WBP, including 8,427 deletions and 356 duplications. Utilizing fixation index (Fst), we determined that 164 CNVs were within the top 1% of the Fst value and defined as under selection. Functional enrichment analyses of the genes associated with these selected CNVs revealed genes linked to reproduction (*SPATA6*, *CFAP43*, *CFTR*, *BPTF*), growth and development (*NR6A1*, *SMYD3*, *VIPR2*), and immunity (*PARD3*, *FYB2*). This study enhances our understanding of the genomic characteristics of the Wanbei pig and offers a theoretical foundation for the future breeding of this breed.

## 1 Introduction

The pig (*Sus scrofa*) was domesticated approximately 10,000 years ago, significantly influencing economic, social, and cultural aspects of human society (1–3). Pig domestication involves phenotypic and genomic alterations, including behavioral changes such as reduced aggression and watchfulness, morphological adaptations like brain size and skull shape alterations, and physiological improvements in growth and prolificacy. To adapt to varying environmental conditions and market demands over different periods, approximately 600 pig breeds have been established worldwide (4). Understanding the relationship between domestication and phenotypic changes is crucial not only for deciphering the genetic basis of complex economic traits but also for advancing future pig breeding practices.

In 2012, a significant milestone was reached in pig genome research with the assembly of the pig reference genome, providing a valuable resource for studying this important livestock species (5). Moreover, the continuous decrease in sequencing costs, driven by technological advancements, has enabled more scientists to explore germplasm characteristics extensively. To date, numerous functional genes and loci regulating important traits have been identified. For instance, the coat color trait, a prominent characteristic in pigs, has been the subject of several studies, identifying genes such as *MITF*, *EDNRB*, *KIT*, and *MC1R* associated with this trait (6–9). Additionally, genes associated with environmental adaptation traits like cold tolerance (*TRPV5*) (10), heat tolerance (*VPS13A*) (11, 12), and hypoxia (*EPAS1*) (13) have been discovered. However, these findings were predominantly based on single nucleotide polymorphism (SNP) detection. Copy number variations (CNVs), ranging from 50 bp to several Mb in size, represent another crucial type of genetic variation in the pig genome (14). The role of CNVs in domestication and their significant impact on phenotypic characteristics, gene function, evolutionary adaptation, and disease susceptibility (15) are less understood. CNVs have been shown to explain complex traits in humans and domesticated animals, such as starch-associated and high-altitude adaptation in humans (16, 17), pathogen and parasite resistance in cattle (18), fatty acid metabolism in dogs (19), and litter size in pigs (20).

The Wanbei pig (WBP), a valuable genetic resource in northern Anhui Province, China, exhibits high fertility, exceptional meat quality, and robust disease resistance. Understanding their germplasm characteristics is vital for their protection and utilization. Previous studies have investigated genomic SNPs in WBP, identifying selected genes based on SNP analysis (21). However, information on CNVs in WBP and selection based on CNVs remains unexplored. Given the significant role of CNVs in elucidating complex phenotypes, detecting CNVs in WBP and understanding their contribution to domestication is essential.

This study utilized resequencing data from 18 WBPs and 19 Asian wild boars (AWBs) to perform a CNV analysis. The study comprises three steps: (1) creating a comprehensive CNV landscape for the Wanbei pig, (2) conducting population structure analysis based on CNVs, and (3) identifying selected CNVs and important candidate genes. The findings of this research enhance our understanding of the impact of CNVs and provide new insights for the protection and utilization of the WBP population.

## 2 Materials and methods

### 2.1 Ethics statement

This study was conducted in accordance with and was approved by the Animal Care Committee of the Anhui Academy of Agricultural Sciences (Hefei, China; no. AAAS2020-04).

### 2.2 Sample collection and whole-genome resequencing

**Figure 1:**
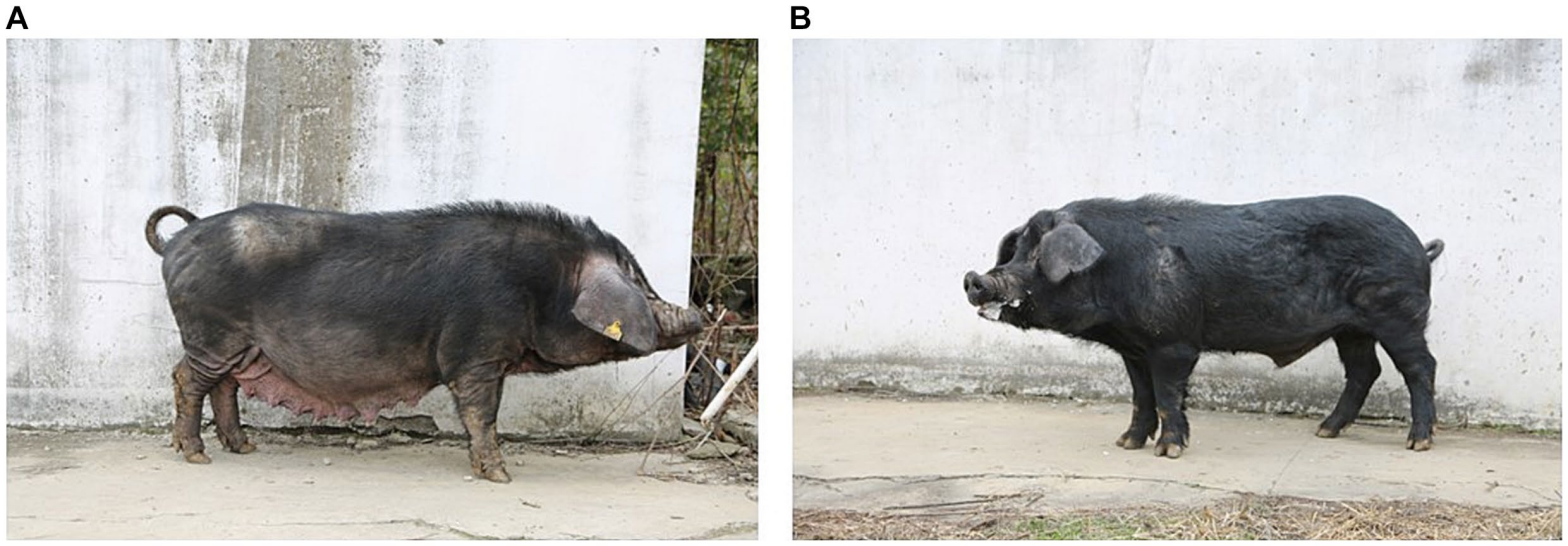
Pictures of Wanbei pig: **(A)** female; **(B)** male.

A total of 18 WBPs (Figure 1), approximately 2 years old, were analyzed through whole-genome resequencing with an average depth of 12.61. The WBPs were sourced from a conservation farm in Yingshang, China (longitude 116.26455E; latitude 32.62893 N). Genomic DNA was extracted from ear tissue samples employing the standard phenol–chloroform extraction method (22). The sequencing library was prepared through a series of steps including random fragmentation of the DNA, purification of the fragments to obtain the desired length, adapter ligation, and DNA clustering. Sequencing was performed on an Illumina NovaSeq 6000 platform (Illumina, San Diego, CA, United States).

To compare population structures and identify selection signatures between WBPs and Asian wild boars (AWBs), we utilized a total of 19 resequenced AWB datasets. Six AWB were sequenced in a previous study, with the data accessible under accession number PRJNA699491 (23). Genomic data for the remaining 13 AWB were retrieved from the National Center for Biotechnology Information (NCBI) under accession numbers PRJNA213179, PRJNA186497, and PRJEB1683, respectively (5, 24, 25).

### 2.3 Read mapping and CNV detection

Prior to CNV detection, adapters and low-quality reads were removed using the NGSQC Toolkit (v.2.30) (26). The filtered reads were aligned to the pig reference genome (*Sus scrofa* 11.1) using the Burrows–Wheeler aligner (BWA) with default parameters. Manta (27) and Paragraph (28) were employed in combination to detect CNVs on autosomes. Specifically, Manta was used for CNV identification and Paragraph for genotyping the variants in each sample. The methods for obtaining high-quality CNVs were based on our previous study (29). PLINK software v.1.90 (30) was used to determine frequency, with visualization conducted using R (v4.2.0).

### 2.4 Principal component and phylogenetic analysis

Before analyzing population structure, VCF files were converted into map and ped formats using PLINK. Two analyses were conducted: principal component analysis (PCA) and phylogenetic trees. PCA was performed using EIGENSOFT (31), phylogenetic trees were created with MEGA v7.0 (32), and visualization was done with ITOL^1^ (33).

### 2.5 Identifying genomic signatures of selection

The fixation index (FST) was calculated to evaluate population differentiation. The formula for Fst calculation is Fst = (Ht − Hs)/Ht, where Ht is the expected heterozygosity of the population, and Hs is the expected heterozygosity of the subgroup. We selected the CNVs at the top 1% of the FST value as signatures of selection. For enrichment analysis of the selected genes within these regions, Gene Ontology (GO) and Kyoto Encyclopedia of Genes and Genomes (KEGG) analyses were conducted using KOBAS.^2^

## 3 Results

### 3.1 Copy number variation identification

To detect genome-wide CNVs and compare the differentiation between WBP and AWB, whole-genome sequencing of 18 WBP and 19 AWB was conducted. The sequencing generated a total of 590.3 Gb for WBP with an average depth of 12.61, and 662.8 Gb for AWB with an average depth of 13.44. Detailed information on WBP and AWB is provided in Supplementary Table S1. Overall, 1253.1 Gb of data were used in this study. We identified 8,783 CNVs in WBP, covering ~30.07 Mb (1.20% of the pig genome), including 8,427 deletions (Del) and 356 duplications (Dup) (Table 1). The average and median lengths of CNVs in WBP are 3,423 bp and 282 bp, respectively (Supplementary Table S2). In AWB, 13,128 CNVs covering 33.32 Mb (1.33% of the pig genome) were detected, including 12,500 Del and 628 Dup (Table 1). The average length of CNVs in AWB is 2,538 bp, and the median length is 259 bp (Supplementary Table S2).

**Table 1:** The statistic of CNV in WBP and AWB.

Population	Total	Number of variants		Total length (bp)/	Length (bp)/Genome ratio	
	Number	Del	Dup	Genome ratio	Del	Dup
WBP	8,783	8,427	356	30,068,462/1.20%	30,045,946/1.20%	22,516/0.001%
AWB	13,128	12,500	628	33,320,072/1.33%	33,278,182/1.33%	41,890/0.002%
Merged	16,408	15,712	696	48,420,217/1.94%	48,373,947/1.93%	46,270/0.002%

**Figure 2:**
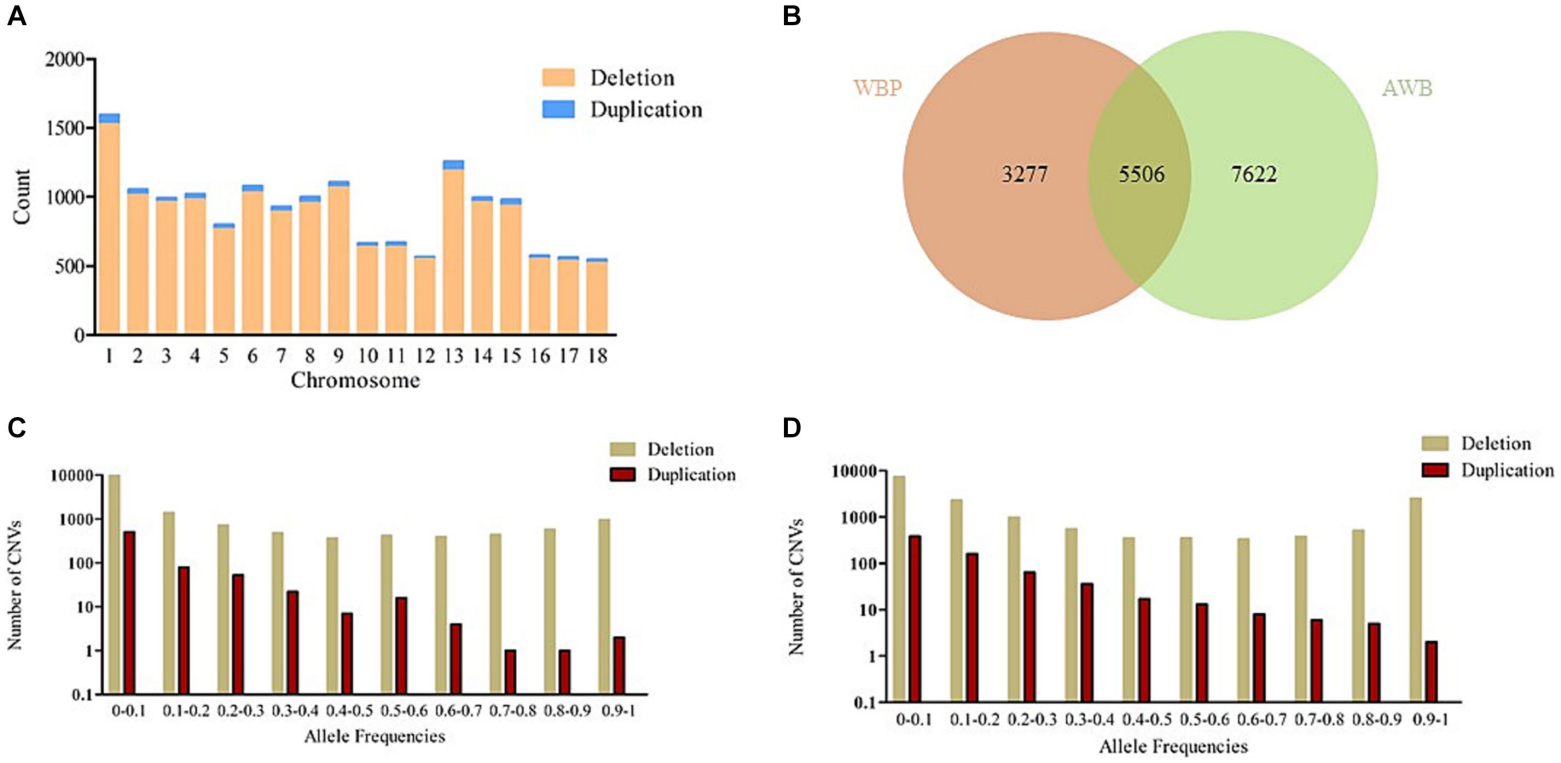
**(A)** The number of CNVs (deletion and duplication) in 18 autosomes. **(B)** The Venn diagram of CNVs between WBP and AWB. **(C)** The allele frequencies of variants in the WBP (*n* = 18). **(D)** The allele frequencies of variants in the AWB (*n* = 19).

After merging the CNVs, a total of 16,408 CNVs (15,712 Del and 696 Dup) were obtained, covering 48.42 Mb of the pig genome, corresponding to 1.94% of the pig genome (Table 1). The information on these CNVs is provided in Supplementary Table S3. The distribution of CNVs across the 18 autosomes varies (Figure 2A). A Venn diagram revealed that 5,506 CNVs (33.56% of the total 16,408) are common between the two populations, and 19.97% are unique to WBP (Figure 2B). Annotation analysis indicated that the CNVs were most abundant in intronic regions (45.75%), intergenic regions (43.44%), and exonic regions (1.23%, 202 CNVs) (Table 2). The frequency of CNVs was divided into ten groups (0–0.1 to 0.9–1), as shown in Supplementary Table S4 and Figures 2C,D. The 0–0.1 frequency group was the largest, covering 63.71% of WBP CNVs and 47.48% of AWB CNVs. The trend between frequency and the number of CNVs in Del and Dup is similar in both populations.

**Table 2:** Annotation of the merged CNVs.

Classification	No. of variants
Downstream	151
Upstream	92
Upstream; downstream	6
Exonic	202
Intronic	7,507
Intergenic	7,127
ncRNA	1,073
Splicing	10
UTR3	191
UTR5	48
UTR5; UTR3	1

### 3.2 PCA and phylogenetic analysis

**Figure 3:**
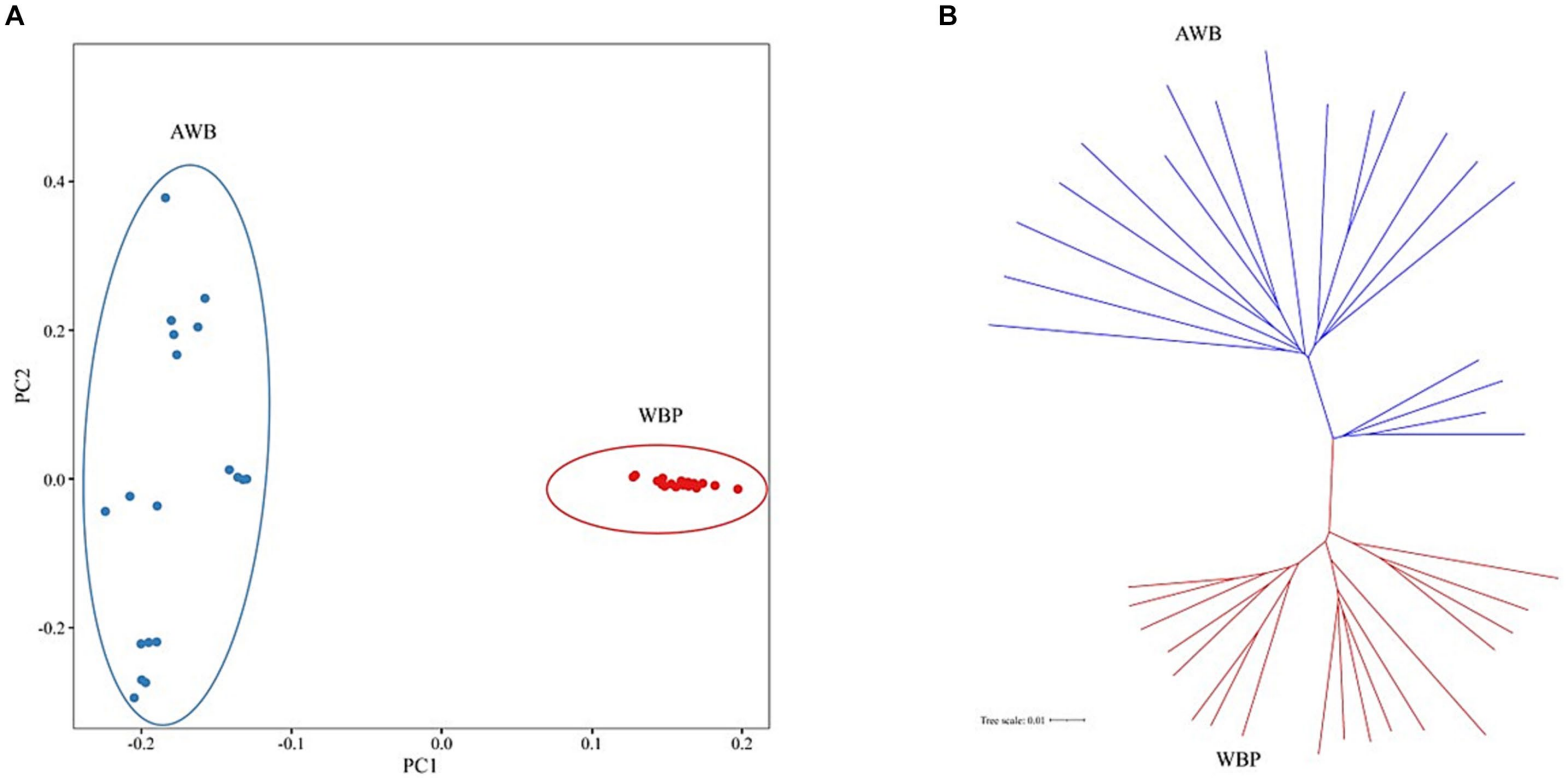
**(A)** PCA plots for the first two PCs for all 37 individuals. **(B)** Neighbor-joining tree constructed from CNV data in study population.

PCA and Neighbor-Joining (NJ) tree analysis were conducted to elucidate the relationship between WBP and AWB populations. Figure 3A shows that PCA, performed with the first two principal components, clustered WBP and AWB separately. A similar pattern was observed in the NJ tree (Figure 3B).

### 3.3 Patterns of selection signatures

**Figure 4:**
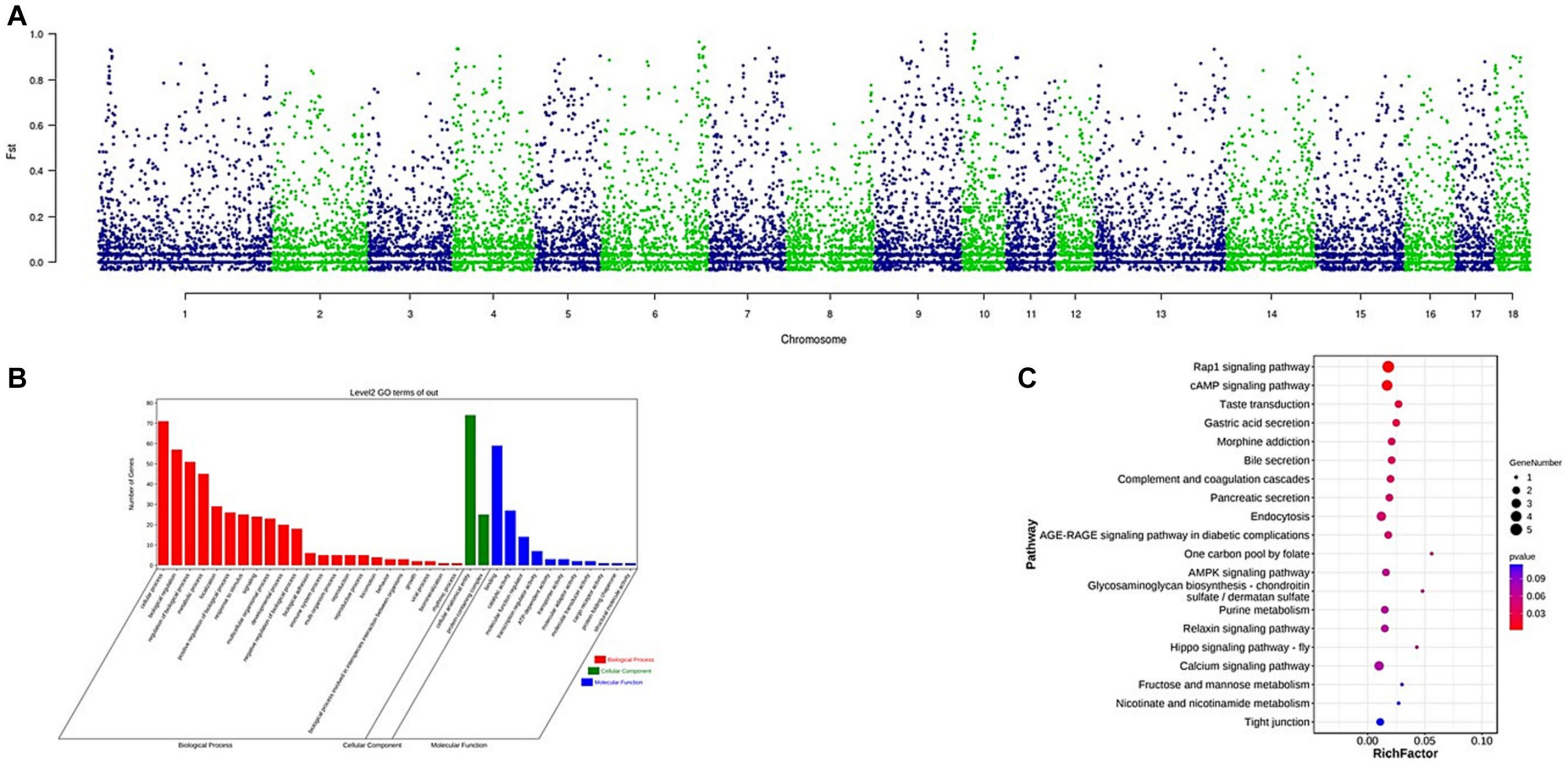
**(A)** Identification of regions with selection in Wanbei pig population compared to Asian wild boar, which are calculated with Fst. **(B)** GO analysis of the selected genes, red referring to biological process, green referring to cellular component and blue referring to molecular function. **(C)** KEGG analysis of the selected genes, the size of dot refers to the number genes related to pathway, and the red to blue indicate the significant value of *p* change.

The Fst was used to identify CNVs under selection across autosomes. A total of 164 CNVs were selected within the top 1% of the Fst value (threshold: 1%, Fst = 0.7741, Supplementary Table S5). The Manhattan plot of the Fst statistic is shown in Figure 4. A total of 84 genes were detected (Supplementary Table S6). Gene ontology (GO) terms and Kyoto Encyclopedia of Genes and Genomes (KEGG) pathways were analyzed using KOBAS software. The GO term analysis enriched a total of 36 terms at level 2 GO enrichment (Supplementary Figure S1; Supplementary Table S7), with these genes associated with growth (GO:0040007, 2 genes), reproduction (GO:0000003, 5 genes), and immune system processes (GO:0002376, 4 genes). The KEGG analysis enriched 10 pathways (Supplementary Figure S2; Supplementary Table S8), including the Rap1 signaling pathway (ko04015, 5 genes), cAMP signaling pathway (ko04024, 4 genes), bile secretion (ko04976, 2 genes), and gastric acid secretion (ko04971, 2 genes).

## 4 Discussion

Pig germplasm resources are national strategic assets and form the foundation for the development of the modern pig industry. As the pig industry evolves towards intensification and large-scale operations, the interest in rearing indigenous pig breeds has diminished significantly. This shift has led to a reduction in the size of local pig populations, a gradual loss of genetic diversity, and the endangerment or even extinction of some local pig breeds. Protecting the genetic diversity of pig breeding resources is imperative, not only to support the future development and utilization of pig breeding but also as a cornerstone for the stable production of pig farming in the future. Additionally, pigs hold significant cultural value for the Chinese people. For instance, the Chinese character for “home” (家) is historically represented by an oracle bone inscription of a pig under a roof, symbolizing treasure and safety. Furthermore, numerous place names in China are associated with pigs, embedding an emotional connection to this animal in every corner of the land. In this study, we analyzed 18 unrelated WBP and 19 AWB to investigate population structure and selection signatures. We detected a total of 8,783 CNVs in WBP and 13,128 CNVs in AWB. Of these, 164 CNVs were under selection, and 84 genes were identified within these selected CNVs. Functional enrichment analysis revealed that the selected genes are associated with several vital traits.

Notably, some genes were linked to reproduction. For instance, the inactivation of Spermatogenesis-associated 6 (*SPATA6*) may lead to sterility (34), while its overexpression has been shown to induce the secretion of testosterone hormone (35). Cilia- and flagella-associated protein 43 (*CFAP43*) is linked with multiple morphological abnormalities of the sperm flagella (MMAF). CFAP43-null male mice were found to be infertile, exhibiting defects in sperm flagella (36–38), and significant associations between *CFAP43* and litter size in goats were identified (39). Cystic fibrosis transmembrane regulator (*CFTR*) plays a role in regulating protein concentration in the reproductive tract (40), and its expression in spermatozoa correlates with sperm quality (41). Lastly, Bromodomain PHD finger transcription factor (*BPTF*) is crucial in establishing the anteroposterior axis of the embryo, with mutations in *BPTF* potentially leading to embryonic death (42). Recent research has indicated that knockdown of *BPTF* is associated with the proliferation and apoptosis of GCs cells (43).

Several genes associated with growth and development were identified. The nuclear receptor subfamily 6 group A member 1 (*NR6A1*) is known to influence the number of lumbar vertebrae (44) and regulate body size in pigs (45). Previous studies have linked *NR6A1* with average daily gain (ADG) and ham weight (46). When comparing CNV positions with PigQTLdb,^3^ 12 quantitative trait loci (QTLs) were identified, including those for ADG (ID = 659, 22,269), body weight (ID = 660, 661, 662), and ham weight (ID = 376). SET and MYND domain-containing protein 3 (*SMYD3*) is crucial in the development of skeletal muscle and myocardium and is involved in regulating myofibril assembly in both muscle types (47, 48). Analysis with PigQTLdb identified QTLs for percentage type I fibers (ID = 7,012, 7,026), and percentage type IIa fibers (ID = 7,034). The vasoactive intestinal peptide receptor 2 (*VIPR2*) gene is associated with insulin secretion (49) and cAMP production (50). Studies have shown that skeletal muscle mass positively correlates with increased cAMP levels upon the administration of a VIPR2-selective agonist (51). *VIPR2* knockout resulted in inhibited growth, reduced fat mass, and increased lean mass (52).

Some genes were identified as immune-related. Porcine epidemic diarrhea virus (PEDV) poses a significant threat to the pig industry. Proteomic analysis comparing PEDV-infected and non-infected groups revealed that *PARD3* is a significantly downregulated protein during virus infection (53). *FYB2* encodes a T cell adaptor protein that can activate integrin and T cell adhesion (54).

Several genes associated with other important traits were also identified. Adenylate cyclase 8 (*ADCY8*), part of the adenylate cyclase family, regulates nutrient homeostasis in rodents (55–57) and has been found under selection in cattle (58) and pigs (21). Previous GWAS studies have linked *ADCY8* positively with Mg and Fe levels (59), high-density cholesterol metabolism in humans (60), and total cholesterol and high-density lipoprotein cholesterol in pigs (61).

However, this study has limitations that should not be overlooked. Due to the limited number of sequenced samples, the detected CNVs may not represent the entire population’s variation. Additionally, the collection of samples to verify the identified CNVs was impeded by the impact of African swine fever. Future plans include collecting a larger sample set to validate the variations and conducting extensive sequencing to more comprehensively detect CNVs.

## 5 Conclusion

In this study, we initially identified the CNVs in the WBP. Subsequently, we analyzed the population structure, comparing WBP with AWB. Furthermore, we identified selection signatures in WBP and discovered several genes associated with reproduction, immunity, growth and development, and lipid metabolism. These insights broaden our understanding of the impact of CNVs in pigs and offer a valuable resource for future genetic breeding endeavors.

## Data availability statement

The data presented in the study are deposited in the Genome Sequence Archive of “China National Center for Bioinformation” (https://ngdc.cncb.ac.cn/gsa/), accession number is CRA015150.

## Ethics statement

The animal studies were approved by the Animal Care Committee of the Anhui Academy of Agricultural Sciences. The studies were conducted in accordance with the local legislation and institutional requirements. Written informed consent was obtained from the owners for the participation of their animals in this study.

## Author contributions

WZ: Conceptualization, Data curation, Investigation, Methodology, Writing – original draft, Writing – review & editing. CX: Resources, Writing – review & editing. MZ: Resources, Writing – review & editing. LL: Resources, Writing – review & editing. ZN: Resources, Writing – review & editing. SS: Resources, Writing – review & editing. CW: Funding acquisition, Writing – review & editing.

## Footnotes

^1^ https://itol.embl.de/

^2^ http://kobas.cbi.pku.edu.cn/

^3^ https://www.animalgenome.org/cgi-bin/QTLdb/SS/index
